# Autoimmune Gastritis With Progression of Leukemic Non-Nodal Mantle Cell Lymphoma

**DOI:** 10.7759/cureus.15762

**Published:** 2021-06-19

**Authors:** Makoto Saito, Masanobu Morioka, Koh Izumiyama, Akio Mori, Takeshi Kondo

**Affiliations:** 1 Internal Medicine and Hematology, Aiiku Hospital, Sapporo, JPN

**Keywords:** autoimmune gastritis, type a gastritis, leukemic non-nodal, mantle cell lymphoma, case report

## Abstract

The pathogenesis of autoimmune gastritis (AIG) remains unclear. In addition, it is difficult to follow the process of AIG onset endoscopically. Leukemic non-nodal mantle cell lymphoma (MCL) was newly added as a subtype of MCL in the fourth revised edition of the World Health Organization (WHO) classification (2017). Here, we report a case of AIG associated with the progression of leukemic non-nodal MCL. A 74-year-old woman who had been followed up in a nearby hospital for chronic B-cell lymphoproliferative disorder with no treatment for six years presented with fever and fatigue in the previous one month. The patient was admitted to our department and was diagnosed with leukemic non-nodal MCL. Positron emission tomography-computed tomography examination, which indicated no abnormalities in the six preceding years, revealed uptake in the bone marrow and spleen. Since MCL was progressing, esophagogastroduodenoscopy (EGD), which showed almost no abnormal findings in the gastric mucosa 13 preceding months, was conducted again to search for lesions involving gastrointestinal MCL. Lymphoma lesions were not found, but wide atrophic mucosal changes in the stomach were revealed mainly in the corpus, and patchy redness was also observed in the pylorus, consistent with AIG. The patient tested positive for an anti-gastric parietal cell antibody (×80), her gastrin level was significantly elevated (5,280 pg/mL), and her pepsinogen (PG) I/PG II was considerably less than 1.0 (>3.1). Although no pathological confirmation was obtained by biopsy, the patient was clinically diagnosed with AIG. In our patient, AIG was revealed to be associated with the progression of leukemic non-nodal MCL in this short period.

## Introduction

Based on the classification of gastritis proposed by Strickland and Mackay in 1973, type-A gastritis produces autoantibodies against the parietal cells of the stomach (anti-gastric parietal cell antibodies), which destroy the fundic glands, resulting in anoxia and hypergastrinemia [1]. Type-A gastritis is now often referred to as "autoimmune gastritis" (AIG) or "autoimmune atrophic gastritis" [2-4] and presents morphologically as atrophic gastritis, mainly in the corpus of the stomach. In addition, it is difficult to follow the process of AIG onset endoscopically.

Meanwhile, the revised version of the WHO classification 2017 added a new disease concept, "leukemic non-nodal mantle cell lymphoma" (MCL), as a subtype of MCL [5]. Unlike classic MCL, this disease has been reported to have an indolent clinical course with unremarkable lymph node lesions, showing peripheral blood, bone marrow, and splenic involvement [5-6]. The acquisition of 17p/TP53 alterations may be a mechanism of tumor progression, which is similar to classic MCL [7].

Here, we report a case of AIG associated with the progression of leukemic non-nodal MCL.

## Case presentation

**Figure 1:**
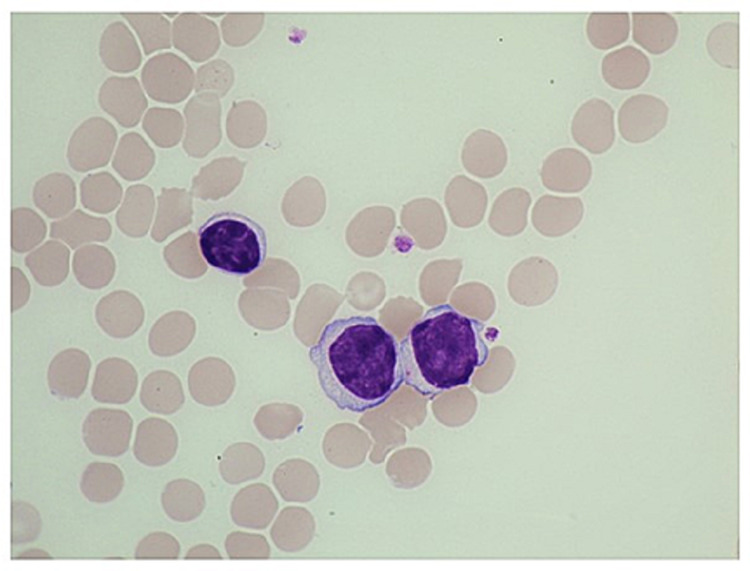
Peripheral blood smear findings Compared to the mature lymphocyte seen above, the two abnormal lymphoid cells are larger and contain nucleoli.

**Figure 2:**
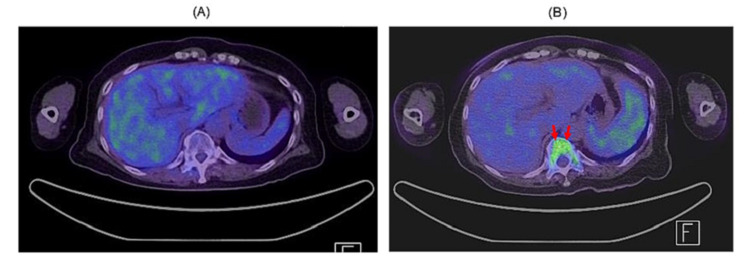
Positron emission tomography-computed tomography findings (A; six years previously, B; current study) (A) No abnormal accumulation was observed. (B) Splenomegaly was observed, and the spleen and vertebral body (bone marrow, red arrow) both had more accumulation (a maximal standardized uptake value ranging from 3.5-5.0) than the liver.

A 74-year-old woman who has been followed for six years since the age of 68 years, without confirmation of a diagnosis of chronic B-cell lymphoproliferative disorder similar to chronic lymphocytic leukemia (CLL), had not received any treatment at a nearby university hospital. She had also been suffering from Parkinson's disease and adenomatous goiter. Her white blood cell (WBC) count, which had been approximately 15,000/μL, had increased to more than 30,000/μL in the last four to five months. Approximately one month previously, the patient developed a fever in the 38°C range, general fatigue, and pain. In addition, positron emission tomography-computed tomography (PET-CT), bone marrow aspiration, and bone marrow biopsy were performed at the hospital, but the diagnosis could not be confirmed. We were delegated to follow up with this patient, who was later hospitalized in our department. The data recorded upon admission are shown in Table 1. Her WBC count was elevated to 55,300/μL, and abundant abnormal, medium-sized lymphoid cells with nucleoli were observed (Figure 1). The abnormal lymphoid cells were positive for the B lymphocyte antigens CD19 (82.8%), CD20 (86.1%), and CD22 (85.1%). CD5 and CD23 levels were negative. Cyclin D1 and p53 deletions were detected (19.1% and 90.0%, respectively) by fluorescence in situ hybridization, and t(11;14) was also confirmed by G-banding chromosomal analysis. A bone marrow biopsy performed at a previous hospital showed overexpression of the p53 protein (not shown). CT imaging showed splenomegaly, but the systemic lymph nodes were not swollen. PET-CT imaging, which showed no abnormalities six years previously, revealed splenomegaly and accumulation in the spleen and bone marrow, with a maximal standardized uptake value ranging from 3.5 to 5.0 (Figure 2). This patient was diagnosed with leukemic non-nodal MCL with p53 abnormalities.

**Table 1:** Laboratory findings on admission Others^#^; abnormal lymphoid cells Abbreviations WBC: white blood cells; St: stab leukocytes; Seg: segmented leukocytes; Mon: monocytes; Eos: eosinophils; Lym: lymphocytes; RBC: red blood cells; Hb: hemoglobin; PLT: platelet; TP: total protein; Alb: albumin; T-Bil: total bilirubin; ALP: alkaline phosphatase; AST: aspartate aminotransferase; ALT: alanine aminotransferase; LDH: lactate dehydrogenase; γ-GTP: γ-glutamyl transpeptidase; CRP: C-reactive protein; sIL-2R: soluble interleukin-2 receptor; AGPA: anti-gastric parietal cell antibodies; AIFA: anti-intrinsic factor antibodies; PG: pepsinogen; *H. pylori*: *Helicobacter pylori*; ANA: antinuclear antibodies; ATMA: antithyroid microsomal antibodies; ATGA: antithyroglobulin antibodies

WBC (4,000-8,000)	55,300	/µL	CD5	13.3	%	TP (6.7-8.3)	5.8	g/dL	AGPA	x80	
St (0-6)	1	%	CD19	82.8	%	Alb (3.8-5.2)	2.4	g/dL	AIFA	(－)	
Seg (45-68)	41	%	CD20	86.1	%	T.Bil (0.2-1.2)	0.9	mg/dL	Gastrin (42-200)	5,280	pg/mL
Mon (2-8)	1	%	CD21	23.0	%	ALP (105-330)	386	IU/L	PG I (> 70.1)	19.6	ng/mL
Eos (0-6)	3	%	CD22	85.1	%	AST (8-38)	86	IU/L	PG II (< 14.9)	20.0	ng/mL
Lym (20-45)	18	%	CD23	0.2	%	ALT (4-44)	41	IU/L	PG I / PG II (> 3.1)	0.98	
Others^#^	36	%	Cyclin D1	19.1	%	LDH (120-245)	792	IU/L	vitamin B12 (180-914)	323	pg/mL
RBC (380-500x10^4^)	350x10^4^	/µL	p53 deletions	90.0	%	γ-GTP (-30)	38	IU/L	*H. pylori *antibody (< 3)	(－)	
Hb (12.0-16.0)	10.4	g/dL				CRP (-0.30)	16.38	mg/dl	ANA (< x40)	(－)	
Plt (12.0-40.0x10^4^)	20.8x10^4^	/µL				sIL-2R (121-613)	2,570	U/mL	ATMA (< x100)	(－)	
									ATGA (< 12.2 IU/mL)	(－)	

**Figure 3:**
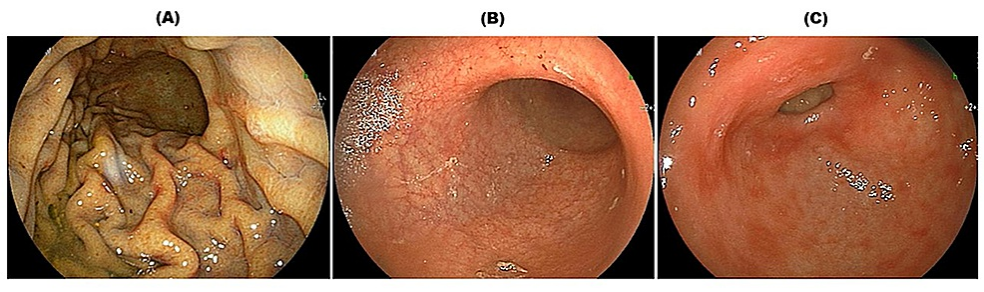
Esophagogastroduodenoscopy findings (A: 13 months previously; B, C: current study) (A) In the corpus of the stomach, the folds of the greater curvature had almost normal morphology, and no atrophic mucosal changes were observed. (B) In the corpus of the stomach, the folds of the greater curvature disappeared, dendritic blood vessels were visible, and a faded atrophic mucosa was observed. (C) In the pylorus, a normal pyloric gland mixed with patchy redness was observed.

Esophagogastroduodenoscopy (EGD) and colonoscopy were performed to identify lesions involving the gastrointestinal tract because MCL easily infiltrates the digestive tract. The patient had undergone EGD 13 months earlier at the university hospital. At that time, almost no abnormalities were observed in the gastric mucosa (Figure 3A). In the current EGD, atrophic mucosal changes were widely observed in the stomach, mainly in the corpus (Figure 3B), but MCL lesions were not found. Patchy redness was also observed in the pylorus (Figure 3C). A biopsy was not performed because we were worried that her general condition would worsen due to bleeding after the biopsy. The patient tested positive for anti-gastric parietal cell antibodies (×80) and negative for anti-intrinsic factor antibodies; she was found to have significantly elevated gastrin (5,280; standard value 42-200 pg/mL), pepsinogen (PG) I (19.6; >70.1 ng/mL), and PG II (20.0 ng/mL) levels, and PG I/II was found to be considerably less than 1.0 (0.98; >3.1). The patient was clinically diagnosed with AIG. Her vitamin B12 levels were normal (323; 180-914 pg/mL), and her anti-Helicobacter pylori antibody was negative (<3 U/mL). In addition, antinuclear and anti-thyroid autoantibodies were negative. The patient had no gastrointestinal symptoms and no history of H. pylori eradication or proton pump inhibitor use.

The patient was treated with ibrutinib for leukemic non-nodal MCL, but it was ineffective and prolonged the period of worsening of her general condition. She subsequently received palliative care at home; endoscopic follow-up of AIG was discontinued.

## Discussion

No histopathological examination was performed on our patient, but since the endoscopic findings and serological features were consistent with AIG [8], this patient was clinically diagnosed with AIG. The patchy redness observed in the pylorus is an endoscopic finding often seen in AIG [9]. The gastric mucosa was almost normal at the first EGD at 13 preceding months, and endoscopic findings of AIG have been clarified during the past 13 months. However, a pathological mucosal change in AIG might have already been present [3-4]. AIG was clearly observed as the leukemic non-nodal MCL had progressed within a short period. It is rare and valuable to be able to follow the onset of AIG with endoscopy.

As AIG progresses, the secretion of intrinsic factors decreases, and pernicious anemia (PA) commonly develops as a complication because the absorption of vitamin B12 is inhibited [10]. In our patient, vitamin B12 levels remained within the normal range, and the onset of AIG was confirmed at an early stage before the onset of complications with PA. In general, AIG is caused by autoimmune cytotoxicity that targets proton pumps (H+/K+ ATPase) in gastric parietal cells [11]; however, its etiology remains unclear. AIG is often associated with other autoimmune diseases, especially chronic thyroiditis [12]; however, it was not found in our patient.

Leukemic non-nodal MCL is a new disease concept and is treated as a subtype of MCL according to the revised 4th edition of the WHO classification (2017) [5]. Patients with indolent leukemic non-nodal MCL may harbor the subclonal p53 mutation, which may serve as a useful biomarker for the early detection of clonal expansion [5-7,13]. In this case, the leukemic non-nodal MCL, which had already developed six years previously, was considered to have progressed because of p53 abnormalities. Although cases of leukemic non-nodal MCL have accumulated [14], no autoimmune disease, including AIG, associated with the progression of non-nodal MCL has been reported. As CLL is often complicated by the presence of autoimmune hemolytic anemia, it should be noted in the future whether leukemic non-nodal MCL is associated with autoimmune diseases.

AIG is also known as the origin of gastric cancer and gastric neuroendocrine cell tumors (NETs) [15-16]. Recently, it was reported that there was a high incidence of gastric cancer and gastric NETs, as well as malignancies in other parts of the stomach, in patients with PA caused by AIG [17]. According to this report, among patients with PA, the risk of developing hematological malignancies is approximately known; the risks of developing multiple myeloma, acute myeloid leukemia, and myelodysplastic syndrome are higher, but non-Hodgkin lymphoma, including lymphoid leukemia, is not high (odds ratio: 1.10; 95% confidence interval: 1.02 to 1.20). Therefore, the development of AIG based on MCL is considered to be extremely rare. Regarding the onset of AIG in the present case, lymphocytes with pathological significance on leukemic non-nodal MCL (i.e., neoplastic cells, nonmalignant B-cells, T-cells, and cellular microenvironment cells) may have been involved in disrupting the maintenance of immune tolerance due to a self-reactive T helper (Th)1/Th2 cell imbalance. This may be a phenomenon that occurred, even in a stable state without the rapid progress of MCL. This time, we thought that after MCL reached complete remission, the improvement/cure of AIG could prove a causal relationship between the two, but unfortunately, that was not possible. In the future, it will be necessary to accumulate numerous similar cases.

## Conclusions

The etiology of AIG remains unclear, and leukemic non-nodal MCL is a new disease concept. In our patient, leukemic non-nodal MCL that had been stable for six years had progressed four to five preceding months. EGD demonstrated a change from almost normal gastric mucosa to AIG in this 13-month period. Here, we report a case in which AIG was confirmed in a short period as leukemic non-nodal MCL progressed. We believe it is valuable to be able to follow the onset of AIG with endoscopy. This patient was suggestive of considering the pathogenic mechanism of AIG.
